# Age Bias in Employment-Related Descriptions: A Word Embedding Analysis of People’s Daily

**DOI:** 10.1093/geroni/igaf122.3699

**Published:** 2025-12-31

**Authors:** Chunyan Mai, Yue Hu

**Affiliations:** The Chinese University of Hong Kong, Hong Kong, China (People’s Republic); The Chinese University of Hong Kong, Hong Kong, China (People’s Republic)

## Abstract

**Background:**

The population is aging globally and older adults’ labor participation is rising. It’s critical to explore factors for post-retirement reemployment. Yet research on the role of aging attitudes is rare. This study investigated age biases in employment-related descriptions in domains of health, personality, skills, employment in People’s Daily from year 1950 to 2021 by exploring whether older age groups are framed as more positively or negatively compared to younger cohorts amid China’s socioeconomic transitions.

**Methods:**

Using natural language processing, word embedding with cosine similarity was applied to analyze associations between age-related words and descriptive words on these people. It was hypothesized that there were periodic fluctuations in age bias across historical stages and stronger associations of younger groups with employment-related words.

**Results:**

Key findings included persistent youth-centric bias, especially post-1980s; fluctuating bias (balanced 1950s–1970s, youth-tilted 1980s–2000s, partially corrected post-2010s); and dimensional differences–health tied to medical resources/aging demands, stable personality perceptions, volatile skills due to industrial shifts, and persistent negative employment bias toward older age groups.

**Discussion:**

Trends reflect adaptations to economic/industrial changes, with societal reliance on youth vitality as a common thread. Differences across domains stem from alignments with age-based values (experience/vitality), illustrating dynamic workplace value adjustments during China’s transitions.

